# Gut dysbiosis as a potential driver of Parkinson’s and Alzheimer’s disease pathogenesis

**DOI:** 10.3389/fnins.2025.1600148

**Published:** 2025-08-13

**Authors:** Jacob M. Pfaffinger, Kallie E. Hays, Jason Seeley, Priyadharshine Ramesh Babu, Rebecca Ryznar

**Affiliations:** ^1^Rocky Vista University College of Osteopathic Medicine, Englewood, CO, United States; ^2^Department of Biomedical Sciences, Rocky Vista University College of Osteopathic Medicine, Englewood, CO, United States

**Keywords:** Parkinson’s disease, Alzheimer’s disease, microbiome, dysbiosis, neurodegeneration, inflammation, short chain fatty acids, lipopolysaccharides

## Abstract

The prevalence of neurodegenerative diseases such as Parkinson’s disease (PD) and Alzheimer’s disease (AD) in the U.S. is expected to increase as the population ages. Despite significant advancements in neurodegenerative research, the initiating events remain unclear, and no treatments currently exist to stop or reverse disease pathogenesis. Emerging studies highlight the importance of the gut microbiome and gut-brain-axis in the pathogenesis of many human diseases. This narrative review aims to integrate current research investigating how gut microbial dysbiosis may influence the development and progression of AD and PD. First, we provide an overview of the pathological features and disease mechanisms characteristic of AD and PD. Next, we summarize existing research on the microbiome–gut–brain axis and how alterations in gut microbiota composition may influence these neurological diseases. We then focus on specific bacterial taxa identified in fecal samples from AD and PD patients, highlighting differences from healthy controls and emphasizing taxa known to produce immunologically relevant metabolites and antigens. Specifically, we examine reductions in short-chain fatty acid (SCFA)-producing bacteria and increases in lipopolysaccharide (LPS)-expressing bacteria that may drive neuroinflammation and contribute to protein misfolding. Finally, this review presents hypothesized mechanisms by which microbial products such as SCFAs and LPS may interact with host physiology to modulate disease pathogenesis. These include pathways involving systemic inflammation, blood–brain barrier permeability, and neural propagation via the vagus nerve or olfactory bulb. Further research is necessary to determine the causes and effects of bacterial level shifts, but understanding the mechanistic roles of these bacterial products in AD or PD pathogenesis could allow for personalized targeted therapies to either slow or potentially reverse the disease process.

## 1 Introduction

Alzheimer’s disease (AD) and Parkinson’s disease (PD) are the two most prevalent neurodegenerative disorders worldwide (National Institute of Neurological Disorders and Stroke, 2023). In the US, nearly 1 million people are living with PD and roughly 6.9 million Americans aged 65 and older have AD. These numbers are predicted to rise as the population ages (Parkinson’s Foundation, 2022; Alzheimer’s Association, 2024). Given the significant societal burden of these disorders, identifying shared mechanisms may provide avenues toward developing broad, effective therapeutic strategies.

AD is a progressive neurodegenerative disease with a slow, insidious onset with a long preclinical phase characterized by progressive abnormal accumulation of beta-amyloid plaques outside neurons and tau tangles inside neurons. This is followed by mild cognitive impairment that progresses to dementia over 10–20 years (Alzheimer’s Association, 2024; Cho et al., 2021; Lloret et al., 2019). In contrast, PD is characterized by the progressive loss of dopamine-producing neurons in the substantia nigra pars compacta due to abnormal alpha-synuclein (*α*-syn) protein aggregation and neuroinflammation (Jankovic, 2008; Fereshtehnejad et al., 2019). Differing to AD, PD often begins with subtle non-motor symptoms such as constipation, olfactory deficits, REM sleep behavior disorder, and autonomic dysfunction, which appear years before the characteristic symptoms of tremors, rigidity, and gait and balance instability become apparent (Jankovic, 2008; Fereshtehnejad et al., 2019). Progression to dementia is typically slower in PD compared to AD, unless a patient presents with combined AD and PD pathology (Sabbagh et al., 2009). Although the onset and progression of symptoms between each disease is unique, each shares similar neuroinflammatory changes and gastrointestinal (GI) symptoms like constipation, suggesting involvement of the gut-brain axis, a bidirectional communication network between the gut microbiome and the central nervous system (CNS) (Abbott et al., 2001; Bridi and Hirth, 2018; Cersosimo and Benarroch, 2012; Hawkes et al., 2010; Li et al., 2023; Shannon et al., 2011; Nedelec et al., 2022). This is supported by numerous studies that showcase significant changes in gut microbiota in PD and AD patients compared to controls (Heravi et al., 2023). Despite emerging findings, few reviews synthesize the distinct microbial alterations in AD and PD within the broader context of their potential impact on systemic and neurological disease mechanisms. Additionally, the utilization of microbiome composition as biomarkers for each disease has not been explored. This narrative review identifies shared and disease-specific pathways and synthesizes current literature to explore how gut microbiome alterations may contribute to AD and PD progression. We aim to highlight potential microbiome-based and therapeutic targets to improve early diagnosis and intervention.

This narrative review aims to synthesize existing literature on gut microbiome alterations in AD and PD, identify overlapping and distinct microbial signatures, and explore the mechanistic links between microbial dysbiosis, neuroinflammation, and disease progression. By integrating neuropathological characteristics, microbiome profiles, fecal metabolite data, and hypothesized microbiota-gut-brain mechanisms, we aim to clarify the role of the microbiota-gut-brain axis in these diseases and highlight novel opportunities for early intervention and targeted treatment.

## 2 Methods

This narrative review synthesizes peer-reviewed studies investigating the role of the microbiome–gut–brain axis in AD and PD, with a particular focus on gut microbiome alterations observed in patients compared to healthy controls. An array of sources, including meta-analyses, clinical studies, and animal models of disease, were reviewed to provide a comprehensive perspective of the current literature. Relevant primary research articles, literature reviews, and meta-analyses were identified using key phrases through databases accessible via Rocky Vista University (RVU), including PubMed, Google Scholar, and the Cochrane Database of Systematic Reviews. Key phrases included: “gut microbiome AND Alzheimer’s disease,” “gut microbiome AND Parkinson’s disease,” “microbiota-gut-brain axis AND (Alzheimer’s OR Parkinson’s),” “gut dysbiosis AND neurodegenerative disease.”

To ensure a comprehensive understanding of the current literature –particularly regarding bacterial metabolites and antigenic factors –an in-depth evaluation of studies comparing fecal samples from AD and PD patients to healthy controls was also conducted prior to drafting the review. During our literature review, any peer-reviewed research that analyzed microbiota levels in AD or PD compared to controls were collected and organized separately. Then, to further isolate these studies into those of interest, we applied inclusion and exclusion criteria to narrow our focus. Studies were included based on the following criteria: only human studies were considered, excluding animal or *in vitro* models; studies had to report bacterial changes at the family, genus, or species level and those reporting only phylum-level changes were excluded; only studies analyzing fecal microbiota samples were included, while those using samples collected via colonoscopy were excluded; a sample size greater than one (n > 1) was required; studies had to include healthy, age-matched control participants; and participants had to have a formal diagnosis of AD or PD based on validated diagnostic criteria. In total, 40 studies met these criteria (9 focusing on AD and 31 on PD) reflecting a greater volume of microbiome research in PD. The outcomes of these studies were organized into a spreadsheet to facilitate comparison of overlapping findings and differences in bacterial composition. Supplementary Tables 1–3 summarize and cite the included studies by taxonomic classification and indicates whether each bacterial group was increased or decreased in abundance compared to healthy controls in the respective study.

While this review highlights selected bacterial changes that appear particularly relevant to AD and PD pathogenesis, it does not aim to comprehensively catalog all microbiome alterations reported in the literature nor perform statistical analysis comparing studies. Instead, the focus is placed on neurological and neuroimmunological changes thought to arise from microbiota-derived metabolites and antigenic factors. Readers interested in a more detailed overview of taxonomic shifts can refer to Supplementary Tables 1–3, which provide additional context and organize relevant studies by specific microbial changes seen in that particular study.

## 3 The pathogenic role of gut dysbiosis in Alzheimer’s and Parkinson’s disease

### 3.1 Overview: pathologic features of PD and AD

To understand how gut dysbiosis may contribute to AD and PD, it is essential to first examine the defining neuropathological features of each condition. For PD, the microscopic pathologic hallmark is the presence of abnormal intracellular aggregates composed of *α*-syn, referred to as Lewy Bodies. These aggregates are often accompanied by Lewy neurites, which are Lewy body-like structures primarily contained within the axons of affected neurons. The protein *α*-syn is a ubiquitously expressed protein within the brain, but in pathologies such as PD and other synucleinopathies, the protein takes on an amyloid-like filamentous structure that is structurally different from the native, non-pathologic form, allowing it to abnormally aggregate and induce the observed clinical symptoms (Kouli et al., 2018). The α-syn protein is the primary component of Lewy bodies, although other proteins have been found in these structures, including ubiquitin, tau, parkin, heat shock proteins, oxidized/nitrated proteins, neurofilaments, MAPs, tubulin, proteasome, and lysosomal elements (Kouli et al., 2018).

AD is also characterized as a protein-conformational disease caused by abnormal processing and folding of normal soluble proteins (Tiwari et al., 2019). In AD, beta-amyloid plaques aggregate extracellularly, while tau proteins form neurofibrillary tangles (NFTs) intracellularly (Tiwari et al., 2019). Beta-amyloid (Aβ, amyloid-beta) misfolding and spread is initiated by inappropriate and incorrect cleavage of amyloid precursor protein (APP), resulting in the formation of amyloid-beta fibrils (Tiwari et al., 2019). These insoluble fibrils then oligomerize into larger aggregates and spread through the nervous system, interfering with synaptic transmission and neuronal signaling (Tiwari et al., 2019). Similarly, tau protein becomes hyperphosphorylated, forming intracellular NFTs. The cause of AD appears to be multifactorial, involving both genetic and environmental factors. Early-onset AD (EOAD), though less common, has a strong genetic basis, with mutations in the amyloid precursor protein (APP) and presenilin (PS1, PS2) genes accelerating amyloid aggregation and disease onset (Lanoiselée et al., 2017). Late-onset AD (LOAD), the more prevalent form, has lower heritability, with the APOE ε4 polymorphism being the most significant genetic risk factor (Yin and Wang, 2018). APOE ε4 is associated with increased amyloid plaque accumulation and neuroinflammation (Yin and Wang, 2018; Dias et al., 2025), and it may influence the gut microbiome in mice, though the extent of this relationship remains under investigation (Zajac et al., 2022).

While *α*-syn makes up the primary component of Lewy bodies in PD, abnormal tau protein aggregation, typically seen in AD, has also been linked to PD. Postmortem studies of many PD patients who develop cognitive dysfunction and dementia report widespread NFTs and beta-amyloid plaques (Pletnikova et al., 2005). These studies show significantly increased levels of hyperphosphorylated tau protein in the striatum of patients (Willa et al., 2010). Animal studies further support the relationship between α-syn and tau, showing that increased α-syn expression can lead to tau hyperphosphorylation *in vitro* and *in vivo* (Duka and Sidhu, 2006; Duka et al., 2006). Beta-amyloid is also reported to act with α-syn in a subset of PD patients, where cortical deposition of α-syn was associated with the formation of beta-amyloid plaques (Lashley et al., 2008). Additionally, current research supports the possibility of dementia symptomology occurring in PD patients due to a convergence of PD and AD pathophysiology in the cortex (Compta et al., 2011; National Institute on Aging, 2017). This overlap in protein aggregation and neurodegeneration suggests that PD and AD may share some common molecular mechanisms, potentially existing on a spectrum rather than as entirely distinct diseases.

Chronic neuroinflammation is a defining feature of both PD and AD, marked by sustained microglial activation and elevated levels of proinflammatory cytokines, which contribute to neuronal dysfunction and accelerate disease progression (Heneka et al., 2015; Hirsch and Standaert, 2021; Tansey et al., 2022). Importantly, this neuroinflammation may be driven not only by intrinsic CNS pathology but also by exogenous factors that induce systemic inflammation. Circulating inflammatory mediators can disrupt blood–brain barrier integrity and interfere with central immunologic homeostasis, further amplifying microglial activation and neuronal injury (Heneka et al., 2015). Given this, focusing on exogenous metabolites or antigenic factors that could promote systemic inflammation may help discover causes of neuroinflammatory processes linked to pathological protein aggregation. Interest then has turned toward gastrointestinal tract pathology as a potential upstream driver of these immune alterations in both PD and AD.

### 3.2 Microbiome and gut-brain axis in AD and PD

In PD, research has identified a range of non-motor symptoms, which can appear years before a clinical PD diagnosis, including loss of smell, depression, sleep disturbances, and constipation (Schenck et al., 1996; Parkinson’s Foundation, 2023). Constipation is the most common nonmotor symptom – affecting over 70% of PD patients – and often precedes motor symptoms by over a decade, with *α*-synuclein aggregates potentially originating in gut submucosal neurons years earlier (Abbott et al., 2001; Bridi and Hirth, 2018; Cersosimo and Benarroch, 2012; Hawkes et al., 2010; Li et al., 2023; Shannon et al., 2011). Despite increasing evidence of these early indicators, diagnosis is typically delayed, and current treatments do not address the underlying pathology.

Although constipation is more strongly associated with PD, it is also observed in AD, where it may appear up to 7 years before clinical diagnosis and persist through disease progression (Nedelec et al., 2022). Studies have found a correlation between constipation and cognitive decline in AD, though the underlying mechanism, such as potential microbiome changes or neuroinflammatory pathways, remains under investigation (Yang et al., 2024).

Constipation does not automatically mean there is dysbiosis or pathology present, but evidence indicates that dysbiosis of gut microbiota may contribute to functional constipation, as well as constipation-type irritable bowel syndrome (Ohkusa et al., 2019). Additionally, constipation in both diseases, especially PD, may be secondary to initial aggregates within the enteric nervous system that impact gut motility, as discussed next.

#### 3.2.1 Gut to brain propagation of misfolded proteins

In PD post-mortem studies, *α*-syn aggregates have been found to have a craniocaudal gradient in the sympathetic chain and gastrointestinal tract, with the most severe pathology in the distal esophagus and stomach, and the least in the rectum (Gelpi et al., 2014). Measurable *α*-syn gradients suggest that pathology spreads from the enteric nervous system to the CNS via the vagus nerve (Gelpi et al., 2014; Braak et al., 2003a). In 2003, the “Braak hypothesis” postulated that Parkinson’s disease pathology may be secondary to neuro-invasion of an unknown pathogen via the olfactory nerve or enteric nervous system. Braak developed a staging system focusing on introductory lesion sites first within the dorsal motor nucleus of glossopharyngeal and vagus nerves, as well as the anterior olfactory nucleus. He hypothesized alpha-synuclein aggregation moved into the brain secondarily (Braak et al., 2003a; Braak et al., 2003b).

The phenomenon of transneuronal propagation—where pathological proteins spread from one neuron to another through the nervous system—has been explored in various neurodegenerative diseases. In mouse models of PD, a study by Kim S. et al. (2019) and Kim Y. H. et al. (2019) supported the hypothesis of transneuronal propagation of *α*-syn from the gut to the brain. It was found that injection of exogenous pre-formed α-syn fibrils (PFF) into the muscularis layer of the pylorus and duodenum stimulated PD-like aggregation and spread of pathologic endogenous α-syn from the myenteric plexus to the hippocampus and prefrontal cortex via the vagus nerve in a time-dependent manner (Kim S. et al., 2019; Kim Y. H. et al., 2019). This led to loss of dopaminergic neurons and PD-like cognitive deficits in the mice (Kim S. et al., 2019; Kim Y. H. et al., 2019). Further supporting the concept of transneuronal spread, both vagotomy and genome excision of endogenous α-syn prior to injection of α-syn PFFs prevented cognitive deficits and PD pathology from developing (Kim S. et al., 2019; Kim Y. H. et al., 2019). This is particularly relevant as human studies have shown that patients with truncal vagotomy have a lower risk of PD than controls (Svensson et al., 2015; Liu et al., 2017).

In Alzheimer’s disease, similar staging mechanisms have been developed with initial tau lesions in the transentorhinal cortex, moving into the entorhinal region and then more neocortical areas (Macedo et al., 2023). The olfactory bulb has been shown to be affected significantly by neurofibrillary tangles and has been considered a potential protein aggregation hub in AD by Braak et al. and others (Braak and Del Tredici, 2015; Mrdjen et al., 2019). This connection may serve as a pathway for protein aggregation, triggered by exogenous exposure through the nasal mucosa and cribriform plate via the olfactory bulb. While no research has yet demonstrated such clear transneuronal propagation, as seen in mouse models of PD, AD studies have highlighted transneuronal spread through early subcortical areas to the limbic and associated cortices (de Calignon et al., 2012). Additionally, a singular autopsy case of Juvenile Alzheimer’s disease highlighted NFTs in the olfactory epithelium (Shimizu et al., 2004), supporting the use of nasal mucosa as target tissue for measuring AD biomarkers (Kim S. et al., 2019; Kim Y. H. et al., 2019).

Signs and symptoms, as well as risk factors for each disease, seem to fit the general location of Braak’s hypotheses of introductory lesion sites (Braak et al., 2003b; Braak and Del Tredici, 2015). Gastrointestinal signs and symptoms in the GI tract, such as constipation, inflammation, and appearance of *α*-syn in colonic submucosa, have been repeatedly reported in PD patients (Braak et al., 2002; Shannon et al., 2012; Devos et al., 2013). Early studies, including Braak et al. (2002) and Shannon et al. (2012) observed intestinal α-syn in aged non-PD individuals and presymptomatic PD patients, respectively. These findings have led to the hypothesis that gastrointestinal involvement may precede central nervous system pathology in Parkinson’s disease. Recent research has further elucidated the role of the gut in PD. For instance, Wang C. et al. (2021) and Wang Q. et al. (2021) highlighted that PD may originate in the gut, where dysbiosis disrupts intestinal barrier integrity and affects neurotransmitter activity. This dysbiosis may lead to increased intestinal permeability, allowing bacterial endotoxins to enter the bloodstream and eventually affect the CNS. However, further research is needed to fully understand the role of the gut in Parkinson’s disease, including the underlying mechanisms and pathways involved. In addition, many insights into the gut-brain axis come from animal models, which may not fully replicate human disease.

#### 3.2.2 GI inflammation in PD and AD

Interestingly, genetic studies suggest that PD patients are more likely to have variations in genes associated with inflammatory bowel disease (IBD) such as Crohn’s Disease, including the leucine-rich repeat kinase genes (Rideout and Stefanis, 2014). However, there are drastic differences between the magnitude of inflammation in IBD (inflammation is visible on colonoscopy) and PD (typically normal on colonoscopy), suggesting that the type of inflammation in these two disorders may be different. In IBD, inflammation is typically characterized by mucosal damage, ulceration, and inflammatory infiltrates (Lee et al., 2021). In contrast, inflammation associated with PD is more subtle and characterized by changes in gut microbiota composition and increase in intestinal permeability (Bellini et al., 2023).

In Alzheimer’s, most research has noticed more proximal GI region inflammation, as there has been a link between periodontal infections and AD (Kurtzman et al., 2024; Jungbauer et al., 2022). One study showed periodontitis exposure is associated with a 1.7-fold increase in the risk of developing AD via retrospective analysis (Chen et al., 2017), and IgG levels to common periodontal microbiota are associated with risk of developing AD (Noble et al., 2014). This may indicate these pathologies were present prior to neurologic damage. Specific periodontal bacteria taxa such as *Porphyromonas gingivalis* can exacerbate neuroinflammation and amyloid-beta deposition (Popescu et al., 2024; Bello-Corral et al., 2023). PD also presents with some proximal GI dysfunction, including gastroparesis and dysphagia (Sharma et al., 2025).

Looking at mid-GI tract areas, specifically the stomach and proximal small intestine, both patients with AD and PD have shown a higher prevalence of positive *H. pylori* testing compared to controls (Huang et al., 2018; Baj et al., 2021; Shen et al., 2017). This is important as *H. pylori* is known to stimulate systemic inflammation, which can disrupt the blood–brain barrier and promote neuroinflammation, potentially contributing to neurodegenerative processes (Bravo et al., 2018). In AD, gastrointestinal changes primarily involve increased intestinal permeability and gut dysbiosis, contributing to systemic inflammation and neuroinflammation (Kuźniar et al., 2024). Distal GI changes in PD present as constipation and colonic mucosal alterations (Sharma et al., 2025).

#### 3.2.3 Neuroinflammation, BBB, and gut permeability

It is known from other diseases that systemic inflammation can worsen neuroinflammation by influencing immunomodulation (Mou et al., 2022; Amanollahi et al., 2023). This is particularly important because neuroinflammation has been consistently defined as a hallmark of both AD and PD (Calsolaro and Edison, 2016; Araújo et al., 2022). Increased pro-inflammatory cytokine densities, such as IL-6, IL-1*β*, TGF-β, and TNF-*α*, have been seen in the serum of AD and PD patients (Lai et al., 2017; Qin et al., 2016) as well as CSF (Chen et al., 2018). Elevated levels of these cytokines can cross the blood–brain barrier, leading to the activation of microglia and astrocytes in the central nervous system, perpetuating a cycle of neuroinflammation and neuronal damage.

Microglia, the resident immune cells of the central nervous system, play a crucial role in neuroinflammation and neurodegenerative changes. M1 microglia are associated with the release of these pro-inflammatory cytokines and contribute to oxidative stress and neuronal damage. In contrast, M2 microglia are involved in anti-inflammatory responses, neuroprotection, and tissue repair (Guo et al., 2022). An increase in M1 microglia activation showed inflammation-mediated degeneration in the enteric nervous system and has been a major focus in neurodegenerative treatment for both diseases (Becker et al., 2018). Gut bacterial dysbiosis may cause systemic activation and subsequent neuroinflammation via M1 microglial activation, altering the normal intestinal immune response and the integrity of the gut barrier and blood-brain barrier (BBB) (Popescu et al., 2024). Furthermore, M2 microglia-activating drugs suppressed neuroinflammation and improved the quality-of-life assessments of PD patients (Wakade et al., 2018).

Interestingly, similar mechanisms of immune activation have been observed in traumatic brain injury (TBI), one of the risk factors for AD and PD (Newcombe et al., 2018; Gardner et al., 2017). Similar to what is seen in PD and AD, major external forces activate M1 microglia, causing an increase in inflammatory cytokines systemically and in the brain. This causes a decrease in blood–brain barrier integrity, contributing to brain pathology due to an increase in systemic immune cell CNS penetration and subsequent worsening of neuroinflammation (Zhang et al., 2018). A meta-analysis by Farrall and Wardlaw (2009) showed a high confidence interval between studies that found Alzheimer’s patients to have increases in BBB permeability compared to controls, and an overall increase in BBB permeability with aging. These immune effects are intuitive when discussing something like TBI due to direct neuronal damage; however, similar effects to the BBB have been observed in gut inflammatory states caused by GI diseases and microbiome dysbiosis (Bravo et al., 2018; Mou et al., 2022).

For example, inflammatory bowel diseases (IBD) led to an increase of peripheral macrophages and inflammatory myeloid cells in the hippocampus (Gampierakis et al., 2020). This suggests that individuals with gut inflammation may experience elevated BBB permeability (Gampierakis et al., 2020). Thus, direct interplay between systemic immunomodulation and neuroinflammation highlights the role of immune cells and cytokines in compromising BBB integrity. It has also been known that the microbiome can regulate systemic inflammation through metabolic pathways, and severe dysbiosis can significantly impact systemic immune regulation and inflammation (Mou et al., 2022; Chen et al., 2022; Thevaranjan et al., 2017). Dysbiosis alters the production of short-chain fatty acids, impairing the function of regulatory T cells and promoting a pro-inflammatory environment (Chen et al., 2022). In addition, dysbiosis increases intestinal permeability, leading to microbial translocation (Thevaranjan et al., 2017). Therefore, gut inflammation due to dysbiosis of bacterial species or improper diet could have major effects on BBB integrity if microglial activation occurs or if the gut inflammation causes a neuroinflammatory state (Shahbazi et al., 2023; Anand et al., 2022; Morys et al., 2024). This could be exacerbated by comorbidities or genetic predispositions to neuroinflammation, the most notable of which being the ApoE4 isoform of the *APOE* gene in AD, which increases the activation of pro-inflammatory M1 microglia (Dias et al., 2025). In PD, genetic mutations in the *PINK1*, *PARKIN*, and *LRRK2* genes have also been associated with inflammatory pathways (Quinn et al., 2020; Sosero and Gan-Or, 2023). As an ongoing area of research, numerous gene loci have been identified as possible risk factors in PD and AD, but their impact on neuroinflammation specifically is not as well characterized (Huang et al., 2022; Jansen et al., 2019; Kunkle et al., 2019). In aggregate pathologies like PD and AD, the increases in neuroinflammation and BBB permeability would ultimately promote the spread of protein aggregates from peripheral nerves into introductory lesion sites in the CNS, as Braak hypothesized (Braak et al., 2003a; Braak et al., 2003b; Braak and Del Tredici, 2015).

### 3.3 Gut microbiota and its relevance to PD and AD

Research supports a bidirectional communication system between the gut microbiota and the brain, recognized as the “microbiota-gut-brain axis.” Bacteria from the phylum Firmicutes and Bacteroidetes form a significant proportion (90%) of the adult gut microbiota, while Actinobacteria composes the rest (O’Toole and Jeffery, 2015). Microbial colonization of the gut occurs during birth, is highly dynamic through infancy, and resembles adult structure by about 3 years of age (Koenig et al., 2010).

The gut microbiota and its antigens and metabolites are directly adjacent to enteric nerves, and alterations in its composition may lead to changes in gut permeability and intestinal barrier function, affecting GI epithelial cells, immune cells, and the enteric nervous system (Di Vincenzo et al., 2024). Although the immune system in the gut normally clears foreign material with minimal inflammation, these changes may provide a means through which bacteria and bacterial products can activate a systemic inflammatory response. As mentioned previously, this systemic immune activation can lead to an impaired blood–brain barrier and may ultimately promote neuroinflammation and worsen neuronal injury and degeneration. At the gut level, production of the pro-inflammatory cytokine TNF-*α* can be facilitated by microbial metabolic pathways, specifically palmitoleic acid metabolism and tryptophan degradation (Schirmer et al., 2016; Hays et al., 2024). Conversely, studies have shown that oral administration of certain “healthy” bacteria such as *Bifidobacterium infantis* have been shown to decrease plasma TNF-α and IL-6 levels (Groeger et al., 2013).

Studies in transgenic mouse models of AD have demonstrated that manipulating gut microbiota can either increase or decrease cerebral amyloid deposition in the brain, indicating some bacterial profiles promote, while others hinder, protein aggregate propagation (Minter et al., 2016; Harach et al., 2017). Similarly, a study in PD model mice confirms that the gut microbiota contributes to motor deficits and neuroinflammation, suggesting that alterations in the human intestinal microbiome may represent a risk factor for PD (Sampson et al., 2016).

A systematic review by Heravi et al. (2023) looked at 42 studies (26 PD and 16 AD) to compare gut microbiota composition in both conditions at the phylum and family levels. Across both healthy and disease groups, the most common bacterial phyla identified were *Bacteroidetes*, *Firmicutes*, and *Proteobacteria*. In PD studies, gut dysbiosis in patients was marked by higher levels of *Akkermansia, Verrucomicrobiaceae*, *Lachnospiraceae*, and *Ruminococcaceae,* while healthy controls had more *Blautia*, *Coprococcus, Prevotellaceae*, and *Roseburia*. In AD studies, *Bacteroides* and *Acidobacteriota* were more abundant in patients, while *Acidaminococcaceae*, *Firmicutes*, *Lachnospiraceae*, and *Ruminiclostridium* were more common in controls. Overall, the microbial signature analysis found associations between PD and bacteria like *Akkermansia*, *Lachnospiraceae, Verrucomicrobiaceae, Bifidobacterium, Ruminococcaceae*, and *Verrucomicrobia*. In contrast, *Ruminococcaceae, Bacteroides,* and *Actinobacteria* were more closely linked to AD (Heravi et al., 2023). While this review is valuable for identifying broad patterns of dysbiosis, its focus on phylum and family levels limits insight into specific microbial products or antigens that may contribute to disease pathogenesis. Although our approach is less formal, our curated sources revealed similar family-level findings (see Supplementary Tables 1, 2). This review builds on that by emphasizing genus-level changes and their potential metabolic or antigenic roles. A summary of these genus-level findings from various studies is provided in Supplementary Table 3 for interested readers.

A prominent example of bacterial alterations reported in literature is the consistent elevation of the genus *Akkermansia* in Parkinson’s disease patients compared to controls (Li et al., 2019; Lin C. H. et al., 2019; Lin C. et al., 2019; Zhang et al., 2020; Bedarf et al., 2017; Heintz-Buschart et al., 2018; Barichella et al., 2019; Vascellari et al., 2020; Keshavarzian et al., 2015; Tan et al., 2021; Baldini et al., 2020; Lubomski et al., 2022; Toh et al., 2022). Although *Akkermansia muciniphila* is generally considered beneficial for gut health, its overabundance in PD may have detrimental effects (Zhao et al., 2024). Notably, some studies have observed an inverse relationship between *A. muciniphila* abundance and the thickness of the gut mucin layer, especially in contexts of prolonged fiber deficiency. This may lead to excessive mucin degradation, resulting in colonic mucosa damage, increased endotoxin leakage, and tissue injury (Desai et al., 2016; Amorim Neto et al., 2022). These context-dependent effects suggest *Akkermansia’s* role can shift from protective to pathogenic under certain conditions, highlighting the need for further research. Moreover, a mouse model demonstrated that *A. muciniphila* can induce mitochondrial calcium overload in enteroendocrine cells, increasing reactive oxygen species (ROS) and promoting *α*-synuclein aggregation within the intestinal mucosa (Amorim Neto et al., 2022). Such findings imply that elevated intestinal *Akkermansia* may contribute to α-synuclein pathology and epithelial barrier dysfunction in PD. Elucidating which microbial metabolites or pathways influence host health will be critical to understanding how the gut-brain axis impacts neurodegenerative diseases like AD and PD.

A key similarity between AD and PD is the potential role of bacterial biofilms in disease progression. Some bacteria, particularly *Escherichia*, release curli amyloid, an extracellular matrix biofilm that has been linked to α-syn aggregation in *C. elegans* and mouse models of PD (Wang C. et al., 2021; Wang Q. et al., 2021; Sampson et al., 2020). Notably, an increase in *Escherichia* populations has been observed, raising the possibility that LPS-induced intestinal permeability could expose endogenous α-synuclein to bacterial biofilms, triggering pathogenic protein aggregation (Zhao et al., 2018; Pietrucci et al., 2019; Vascellari et al., 2020; Hopfner et al., 2017; Toh et al., 2022; Cattaneo et al., 2017.) Given that bacterial components have also been found in AD plaques (Miklossy, 2016; Dominy et al., 2019), a similar mechanism may contribute to amyloid pathology in AD. Additional microbial shifts related to specific metabolites and antigenic factors are discussed in subsequent sections.

It is important to note here that most current evidence in humans derives from observational studies, which can demonstrate correlations between dysbiosis and neurodegenerative pathology but are limited in establishing causality. These studies often face confounding variables related to host physiological differences and significant microbial variation between individuals, even within the same population. Also, utilizing humans in experimental models that could provide some insight into causality—such as fecal microbiota transplantation from diseased individuals to healthy ones—would be unethical. Therefore, it is vital to interpret all studies within their respective limitations, acknowledging that establishing a direct causal relationship between microbial products and neurodegeneration will likely depend on animal disease models, which pose challenges in translating findings to humans.

#### 3.3.1 Short chain fatty acids in AD and PD

Much of the microbiome’s effects on host homeostasis are due to short-chain fatty acids (SCFA), metabolites derived from bacterial fermentation of dietary fibers and polysaccharides in the gut that have been shown to fuel intestinal epithelial cells and regulate their function (Parker et al., 2020; Parker et al., 2020; Martin-Gallausiaux et al., 2021). Acetate, butyrate, and propionate are the primary SCFA molecules produced from gut bacteria by fermentation (Mann et al., 2024). These SCFAs play crucial roles in maintaining intestinal homeostasis, modulating immune responses, and supporting gut barrier function (Yamada et al., 2015; Silva et al., 2020; Hays et al., 2024). Among them, butyrate is particularly well-characterized due to its robust anti-inflammatory properties and neuroprotective effects (Hays et al., 2024; Singh et al., 2014).

In patients with Parkinson’s Disease (PD), quantitative analysis of fecal samples has revealed significantly reduced concentrations of acetate, propionate, and butyrate compared to healthy controls (Unger et al., 2016). These reductions exceeded the typical age-related decline, suggesting a disease-specific alteration in SCFA metabolism. These findings support the hypothesis that reduced butyrate levels may impact the enteric nervous system (ENS), potentially contributing to gastrointestinal dysmotility—a common non-motor symptom of PD (Unger et al., 2016).

In Alzheimer’s disease (AD), findings have been more heterogeneous. Some studies report increased salivary concentrations of acetate and propionate but decreased butyrate levels in both feces and brain samples (Iilmaz et al., 2017; Zhang et al., 2017). Yet other data show lower serum acetate concentrations in AD patients, highlighting the complexity and inconsistency in SCFA distribution across body compartments (Cui et al., 2020).

Mechanistically, butyrate plays a uniquely protective role among SCFAs. It has been shown to reduce pro-inflammatory cytokine release from dendritic cells and protect dopaminergic neurons, whereas these effects have not been as clearly demonstrated for acetate or propionate (Hays et al., 2024; Nastasi et al., 2015; Kidd and Schneider, 2010). In mouse models of TBI, butyrate treatment improved neurological function, decreased brain edema, and mitigated neurodegeneration and BBB dysfunction (Li et al., 2018). It has also been shown to enhance *α*-synuclein clearance, reduce amyloid uptake, and improve cognition and memory in PD and AD models (Qiao et al., 2020; Marizzoni et al., 2020; Wang et al., 2022). These findings support the importance of butyrate in neurodegeneration prevention due to its potential to impact inflammation and protein aggregation.

Genus-level analyses in both PD and AD cohorts have demonstrated reductions in known SCFA-producing bacteria, including *Blautia, Roseburia*, and F*aecalibacterium* (Lin et al., 2018; Zhao et al., 2018; Vascellari et al., 2020; Keshavarzian et al., 2015; Scheperjans et al., 2015; Tan et al., 2021; Hopfner et al., 2017; Liu et al., 2018; Bedarf et al., 2017; Weis et al., 2019; Cosma-Grigorov et al., 2020; Cirstea et al., 2020; Haran et al., 2019; Toh et al., 2022; Hill-Burns et al., 2017; Petrov et al., 2017; Lubomski et al., 2022; Li et al., 2017; Ueda et al., 2021). These genera are among the most consistent SCFA producers, and their decline may lead to reduced SCFA levels seen in feces and serum.

While it is challenging to attribute specific SCFA production to genera due to intra-genus variability, several reviews estimate SCFA preferences by examining metabolic pathways (Hays et al., 2024; Deleu et al., 2021). For instance, *Roseburia* and *Faecalibacterium* are more closely linked to butyrate production (Hays et al., 2024). In support of this, the species *Roseburia intestinalis* has demonstrated anti-inflammatory activity in colitis models, presumably due to its butyrate output (Ruan et al., 2022). Loss of such butyrate-producing bacteria may therefore contribute to gut inflammation and compromised intestinal barrier function in neurodegenerative conditions.

Conversely, changes in *Blautia* levels may exert different effects. Although often cited as a SCFA producer associated with acetate and propionate, increased levels of *Blautia* have been implicated in metabolic disorders, potentially due to acetate-driven insulin release (Ordoñez-Rodriguez et al., 2023). In studies on multiple sclerosis, *Blautia* levels were inconsistently altered, with some reporting increases and others decreases (Aresella et al., 2020; Ventura et al., 2019). This suggests *Blautia*’s role in disease may depend on the disease context or other microbial interactions.

Despite butyrate’s extensive characterization, recent reviews have indicated that acetate and propionate may also confer neuroprotective benefits in the context of AD and PD (Popescu et al., 2024; Alam et al., 2024; Yassin et al., 2025). Interestingly, although SCFA-producing bacteria are frequently altered in AD and PD, those increases often favor acetate and propionate production. Still, *Blautia*, a key producer of these acids, is reduced in many PD cohorts. This suggests that there may be optimal ranges for SCFA concentrations, and excesses or deficiencies may both contribute to disease risk. Targeted profiling of each SCFA’s role is necessary for deeper mechanistic insight. Together, these findings suggest that SCFA imbalances—particularly reduced butyrate and context-dependent shifts in acetate and propionate—may disrupt gut and brain homeostasis in PD and AD, supporting a key mechanistic link between microbial metabolism and neurodegenerative disease progression.

#### 3.3.2 Other metabolites associated with PD and AD

Vascellari et al. (2020) correlated microbiome changes with metabolic alterations by using gas chromatography–mass spectrometry (GC–MS) to analyze gut metabolites in PD patients at a family level. Their findings showed an increase in *Bifidobacteriaceae* corresponded with decreased pyroglutamic acid and glutamic acid, and increased *Bacteroidaceae* in PD patients was correlated with decreased linoleic acid. Increased *Streptococcaceae* and decreased *Sphingobacteriaceae* were both linked to higher cadaverine levels. No similar study was done in AD patients.

Glutamic acid is a neurotransmitter implicated in PD and AD pathogenesis (Figura et al., 2018; Chang et al., 2020). As a precursor of glutathione, reduction in glutamic acid may reflect an increase in oxidative stress in disease progression or secondary to microbial shifts that impact glutamate metabolism (Chang et al., 2020). Also, several studies have reported that the serum of PD patients showed decreased levels of several long-chain omega-6 polyunsaturated fatty acids (PUFAs), including linoleic acid (Hernando et al., 2019; Schulte et al., 2016). Like glutamic acid, linoleic acid has been associated with protective effects against oxidative stress, thus suggesting that reduction in PUFAs in PD may reflect excessive oxidative stressors leading to increased disease progression (Vascellari et al., 2020). Conversely, AD derived gut microbiota in a study of mice was shown to enhance proinflammatory pathways for PUFA metabolism in the brain (Chen et al., 2022). Thus, increased PUFAs in the gut could worsen AD pathogenesis due to microbiome dysbiosis. Interestingly, oxidized linoleic acid metabolites (OXLAMs) have been associated with AD pathogenesis, thus there could be negative effects to the oxidation process as well (Mercola and D’Adamo, 2023). Therefore, in both AD and PD, decreased PUFAs might actually reflect increased oxidative damage leading to the harmful oxidation products that worsen neurodegeneration.

The polyamine cadaverine is a product of bacterial and human co-metabolism and has been reported to have a toxic effect, likely due to oxidative stress produced by its catabolism (Vascellari et al., 2020; Santoru et al., 2017). Some studies suggest that cadaverine is involved in the inhibition of intestinal motility (Sanchez et al., 2017; de la Rivas et al., 2006), which may decrease the normal clearance of pathogens or toxic substances from the GI tract. Another study found an increase in cadaverine in patients with IBD, suggesting that higher levels of cadaverine may be associated with a proinflammatory environment (Santoru et al., 2017). The risk of periodontitis and AD may be facilitated by an increase in cadaverine due to its ability to interfere with cell signaling and cause leukocyte migration disruption (Amin et al., 2021).

#### 3.3.3 Lipopolysaccharide and inflammatory pathways in AD and PD

Gram-negative bacteria represent a significant public health concern due to their high antibiotic resistance and frequent involvement in human disease (Oliveira and Reygaert, 2022). Among them, certain genera commonly found in the human gut, such as *Alistipes* and *Bacteroides*, have been increasingly implicated in inflammatory and neurodegenerative conditions. *Alistipes*, a relatively new genus within the Bacteroidetes phylum, has been associated with gut inflammation and psychological disorders including anxiety, depression, and myalgic encephalomyelitis/chronic fatigue syndrome (Parker et al., 2020; Parker et al., 2020). Several studies have reported increased abundance of *Alistipes* in both PD and AD (Lin et al., 2018; Li et al., 2019; Qian et al., 2018; Qian et al., 2020; Li et al., 2020; Toh et al., 2022; Vogt et al., 2017; Cammann et al., 2023; Haran et al., 2019; Hung et al., 2022). Similarly, *Bacteroides* has been frequently identified in inflammatory contexts. *Bacteroides vulgatus* and *Bacteroides fragilis*, for instance, have been isolated from patients with Crohn’s disease and are associated with intra-abdominal abscesses, appendicitis, and inflammatory bowel disease (Zafar and Saier, 2021). In AD and PD patients, *Bacteroides* shows variable trends, with some studies reporting increased abundance (Keshavarzian et al., 2015; Vogt et al., 2017; Cammann et al., 2023; Haran et al., 2019; Hung et al., 2022) and others showing reductions (Vascellari et al., 2020; Petrov et al., 2017; Zhuang et al., 2018).

**Figure 1:**
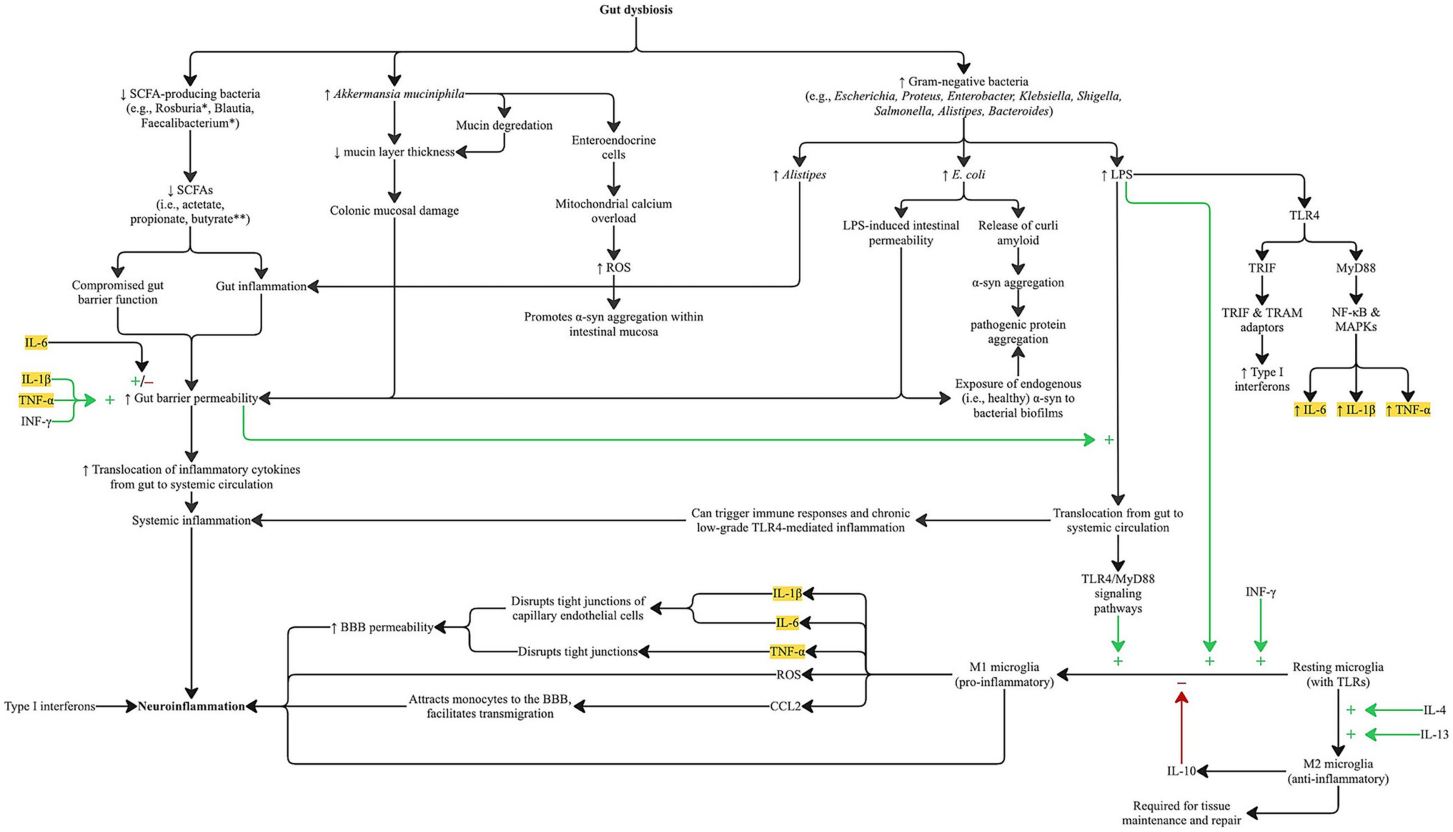
Pathways by which gut dysbiosis associated with AD and PD promotes gut and neuroinflammation through systemic and microglial-mediated cytokine release. Disease-specific alterations in gut microbial composition differentially affect signaling cascades but converge on increasing gut barrier permeability via elevated proinflammatory cytokines commonly observed in both AD and PD. Notably, lipopolysaccharide (LPS) from specific gut bacteria activates TLR4/MyD88 signaling, leading to increased production of TNF-α, IL-1β, and IL-6 through the MyD88-dependent pathway, and type I interferons via the TRIF-dependent pathway. Locally, gut-derived cytokines disrupt epithelial integrity and enhance permeability, while systemically they drive chronic inflammation. Gut barrier dysfunction may be further exacerbated by reduced levels of SCFAs and increased mucin degradation, particularly from *Akkermansia muciniphila*. TLR4/MyD88 activation also contributes to M1 microglial polarization and CNS cytokine release, which compromise tight junctions in cerebral endothelial cells, thereby increasing BBB permeability. These inflammatory pathways may also contribute to pathological protein aggregation. Legend:* Bacteria closely linked to butyrate production. **Bacteria with robust anti-inflammatory and neuroprotective properties. Highlighted cytokines are those elevated in the serum and CSF of AD and PD patients.α-syn: alpha-synuclein; BBB: blood–brain barrier; LPS: lipopolysaccharide; ROS: reactive oxygen species; SCFA (s): short-chain fatty acid(s); TLR: toll-like receptor; TRIF: TIR-domain–containing adaptor-inducing interferon-β; TRAM: TRIF-related adaptor molecule.

The Enterobacteriaceae family, accounts for approximately 80% of gram-negative isolates and includes several other pathogenic genera such as *Escherichia*, *Proteus*, *Enterobacter*, *Klebsiella*, *Citrobacter*, *Yersinia*, *Shigella*, and *Salmonella*. A key structural component of these bacteria, shared with *Alistipes* and *Bacteroides,* is lipopolysaccharide (LPS), a potent endotoxin. At the gut level LPS activates TLR4 receptors that lead to a rapid release of pro-inflammatory cytokines, including TNF-*α*, IL-1β, and IL-6, via both MyD88-dependent and TRIF-dependent pathways, resulting in increased intestinal permeability, immune cell recruitment, and amplification of local and systemic inflammation (Gryka-Marton et al., 2025; Singh et al., 2022; Guo et al., 2015; Bruning et al., 2021). Figure 1 illustrates the pathways through which gut dysbiosis, driven by specific microbiota shifts observed in AD and PD, induces distinct patterns of cytokine release. The MyD88-dependent pathway is activated at the plasma membrane upon TLR4 engagement of LPS leading to the activation of NF-κB and MAPKs, resulting in the release of pro-inflammatory cytokines including TNF-*α*, IL-1β, and IL-6. The TRIF-dependent pathway is activated after TLR4 is internalized into endosomes, where it recruits TRAM and TRIF adaptors producing type I interferons and a delayed wave of pro-inflammatory cytokines, likely contributing to chronic inflammation and further immune cell recruitment (Perez-Pardo et al., 2019; Lin C. H. et al., 2019; Lin C. et al., 2019). When translocated from the gut into systemic circulation, LPS can trigger immune responses systemically and chronic low-grade TLR4-mediated inflammation as well (Di Vincenzo et al., 2024; Wei et al., 2024). This process, often referred to as metabolic endotoxemia, has been implicated in the development of type 2 diabetes, multiple sclerosis, and neurodegeneration in mouse models (Cani et al., 2008; Gambuzza et al., 2011). LPS may also prime microglia through TLR4/MyD88 signaling pathways, further amplifying neuroinflammation via further cytokine release (Wang et al., 2023; Ausseil et al., 2008).

**Figure 2:**
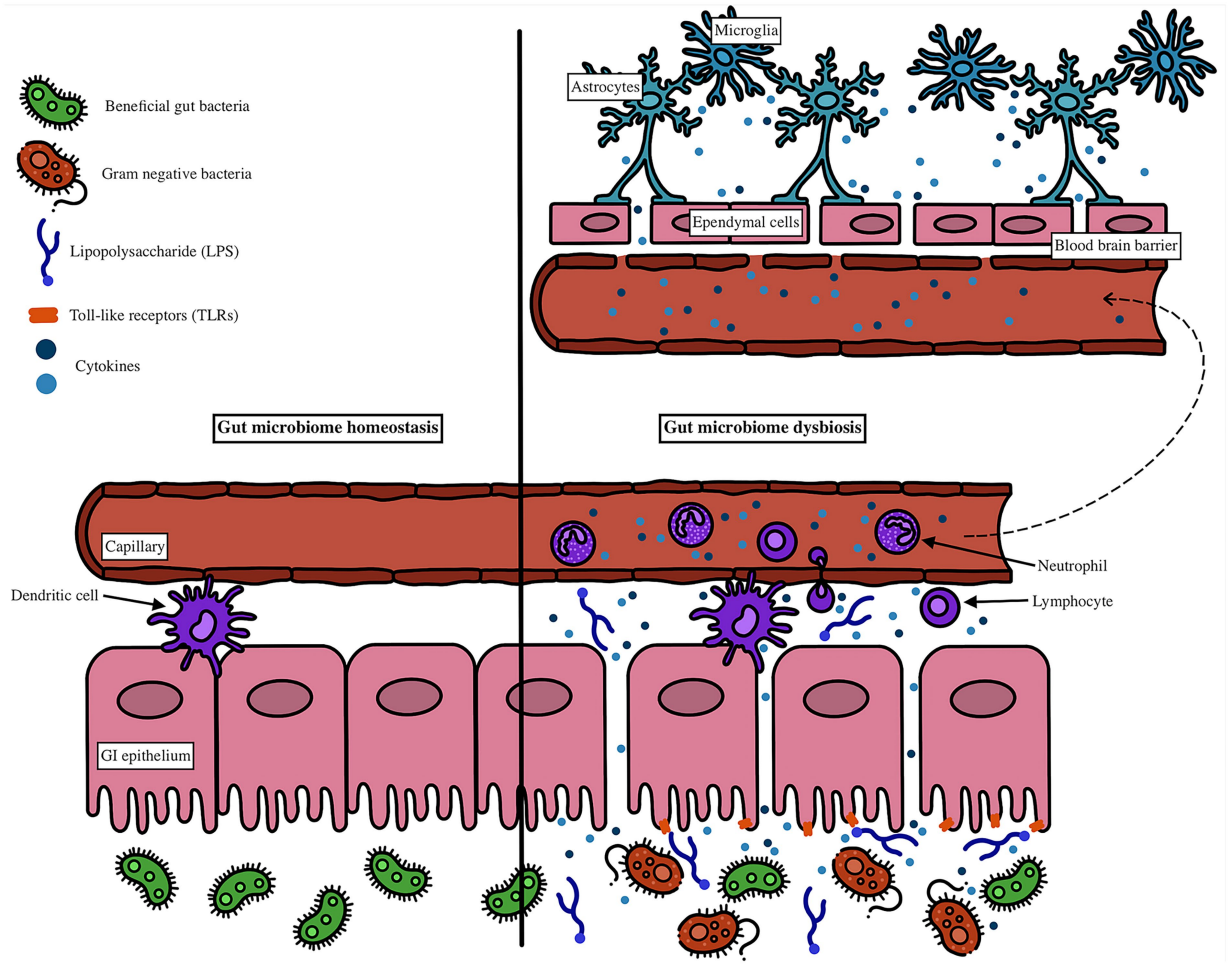
Lipopolysaccharide (LPS) binds to Toll-like receptor 4 (TLR4) on gut epithelial and immune cells, including dendritic cells, triggering the release of proinflammatory cytokines. Activated dendritic cells amplify the immune response and promote systemic inflammation as cytokines enter circulation. These circulating mediators reach the brain, where they activate microglia and astrocytes, leading to the release of additional proinflammatory factors that disrupt neuronal function. Astrocytes also contribute to blood–brain barrier (BBB) breakdown, while ependymal cells, normally involved in cerebrospinal fluid regulation, respond to inflammatory signals and may facilitate further neuroinflammatory spread. This cascade increases BBB permeability, allowing more immune mediators into the CNS and exacerbating neuronal dysfunction.

LPS from Gram-negative bacteria can also activate TLR4 on immune cells such as dendritic cells (Banks et al., 2015). Activated dendritic cells contribute to both local and systemic immune activation by amplifying cytokine production and trafficking to lymphoid tissues. Once in circulation, these cytokines can affect distant organs, including the brain. Within the central nervous system, these circulating inflammatory mediators can promote activation of microglia and astrocytes, both of which secrete additional inflammatory factors that impair synaptic function and neuronal health (Erny et al., 2015; Ransohoff and Brown, 2012). Astrocytes also play a role in compromising BBB permeability, further enabling immune mediators to infiltrate the brain parenchyma (Braniste et al., 2014). In parallel, ependymal cells lining the brain ventricles, which regulate cerebrospinal fluid homeostasis, respond to these inflammatory cues and may facilitate neuroimmune signaling. The culmination of these processes is a feed-forward loop of neuroinflammation, increased BBB permeability, and progressive neuronal dysfunction (Engelhardt and Ransohoff, 2012). These interactions at the gut epithelium and blood–brain barrier are illustrated in Figure 2.

Increased levels of *Escherichia/Shigella* have also been reported in multiple studies of AD and PD (Zhao et al., 2018; Pietrucci et al., 2019; Vascellari et al., 2020; Hopfner et al., 2017; Toh et al., 2022; Cattaneo et al., 2017). These genera are associated with gut-brain axis pathology, including generalized anxiety disorder (Baske et al., 2024). While these increases in LPS-producing bacteria support the idea of LPS-induced inflammation, recent findings complicate this interpretation. Some commensals produce structurally distinct forms of LPS with lower immunogenicity, particularly due to differences in the lipid A domain, which can reduce TLR signaling and induce anti-inflammatory effects (d’Hennezel et al., 2017).

LPS itself is highly variable. Its structure can differ by genus, species, and even strain, and bacteria vary in how much LPS they produce. These differences contribute to diverse immune outcomes (Caroff et al., 2002). For example, *Shigella flexneri* LPS is about 100 times less immunostimulatory than *E. coli* LPS, whereas *Pseudomonas testosteroni* LPS is 60 percent more active (Laude-Sharp et al., 1990). These observations likely reflect evolutionary adaptations in commensal and pathogenic bacteria (Caroff et al., 2002).

This variability suggests that the inflammatory consequences of LPS depend not just on abundance but also on the types of LPS present. A shift toward highly immunogenic LPS-producing species may exacerbate systemic inflammation and neurodegenerative disease progression. Species-level analysis, especially focusing on the immune activation potential of LPS variants, could provide deeper insight into microbial contributions to neuroinflammation in AD and PD.

It is also important to consider intra-genus variability. *Alistipes*, *Escherichia/Shigella*, and *Bacteroides* are all gram-negative and capable of expressing LPS, but their impact likely varies based on species-specific antigens and metabolites. Elevated bacterial loads may enhance TLR activation, but additional factors such as LPS subtype or metabolic byproducts may shape the host immune response. Future research should aim to identify species and strains whose LPS profiles or associated metabolites directly contribute to neuroinflammatory states or pathological protein aggregation in AD and PD.

##### 3.3.3.1 LPS and protein aggregation in both AD and PD

*In vitro* and *in vivo* studies have demonstrated an association between LPS and the pathologic protein changes seen in AD and PD, discussed earlier. In AD, LPS has been found within the typical senile plaque lesions of AD brains (Asti and Gioglio, 2014; Sheng et al., 2003). Interestingly, co-incubation of Aβ peptide with *E. coli* LPS organized compact fibrils and potentiated amyloid fibrillogenesis (Asti and Gioglio, 2014). Also, systemic injection of LPS in wild-type and transgenic AD mice results in greater amyloid deposition and tau pathology compared to saline-injected controls (Sheng et al., 2003).

In PD, LPS is known to modulate *α*-syn aggregation *in vitro* and elicits multiple PD-like pathological effects in vivo (Liu and Bing, 2011; Paslawski et al., 2014). LPS accelerates α-syn aggregation kinetics by increasing growth rate and decreasing half-life and lag time; this is associated with the formation of alternative intermediate conformations that dictate the overall fibrillar morphology (Paslawski et al., 2014). Wild-type α-syn alone undergoes a gradual structural conversion from random coil to beta-sheet structure via formation of a transient, helix-rich intermediate state that gives way to matured beta-sheet fibrillar forms (Paslawski et al., 2014). However, LPS and α-syn interactions result in stabilization of these intermediate helical forms (Liu and Bing, 2011). In one study, these intermediates remained stable for about 96 h when equimolar concentrations of LPS:α-syn were used, and the duration of LPS-mediated stability increased with increasing concentrations of LPS (Bhattacharyya et al., 2019).

Thus, the role of the gut microbiota in neurodegenerative diseases likely extends beyond general inflammation, involving specific microbial profiles that may actively promote or inhibit protein aggregation and neuroinflammation. However, definitively establishing a causal relationship between microbial products, such as LPS, and neurodegenerative pathology remains challenging. This difficulty is compounded by the limited translatability of animal models to human disease, as well as the high variability in human microbiota composition, immune thresholds, and baseline gut epithelial integrity, all of which introduce significant confounding variables.

### 3.4 Hypothesized microbiota-gut-brain axis mechanisms for PD and AD pathogenesis

**Figure 3:**
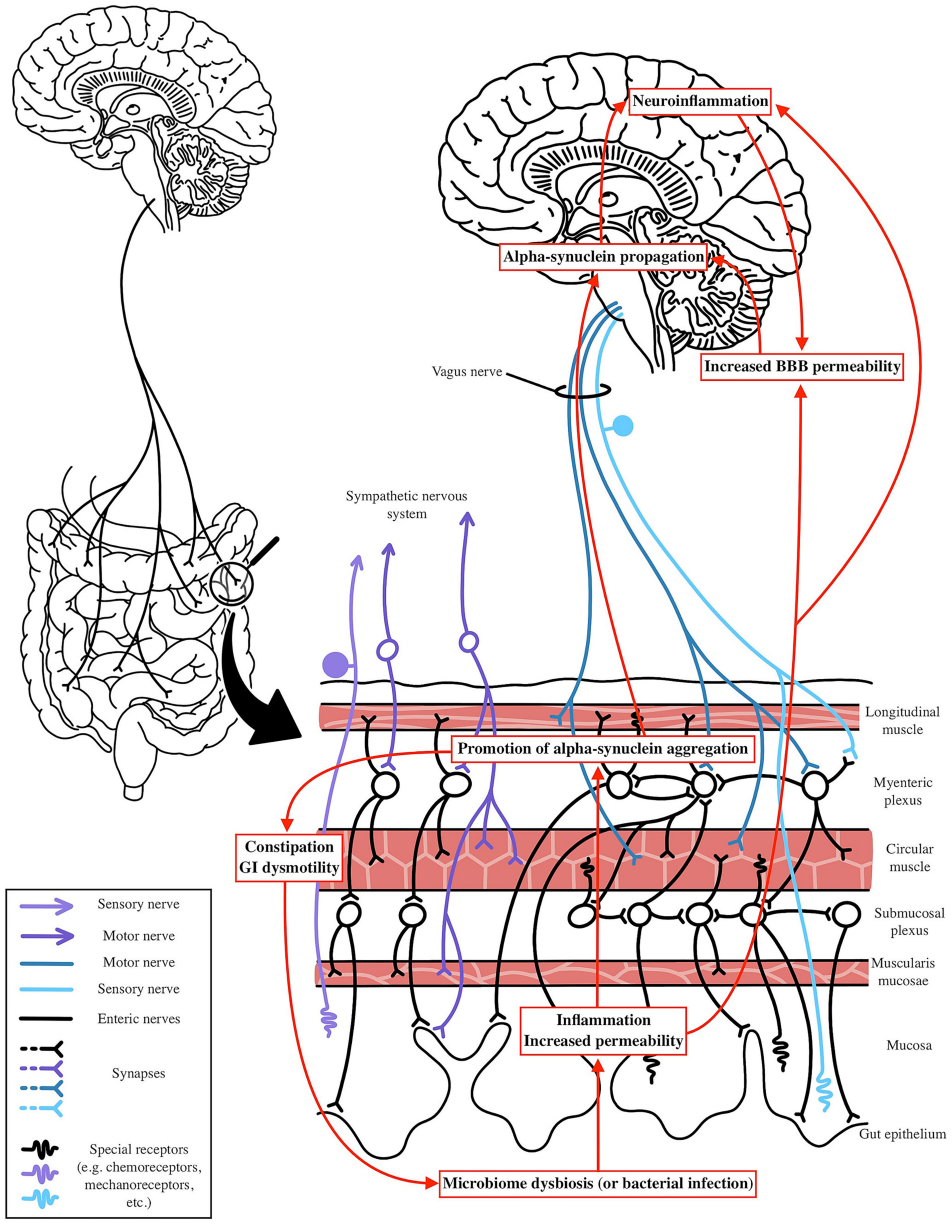
Potential mechanism by which PD is impacted by microbiome shifts. Microbial dysbiosis or infection can elevate gut and systemic inflammation through LPS-induced inflammatory pathways and reduced SCFA production. This systemic and neuroinflammation compromises BBB integrity, facilitating alpha-synuclein propagation via the Vagus nerve to the CNS. Dysbiosis may trigger gut-level alpha-synuclein aggregation in response to exogenous factors, with systemic inflammation further enhancing its spread. In the gut, alpha-synuclein aggregation disrupts enteric nervous system function, leading to constipation and GI dysmotility, which further exacerbates microbiome dysbiosis, creating a vicious cycle of neuroinflammation and gut dysfunction.

**Figure 4:**
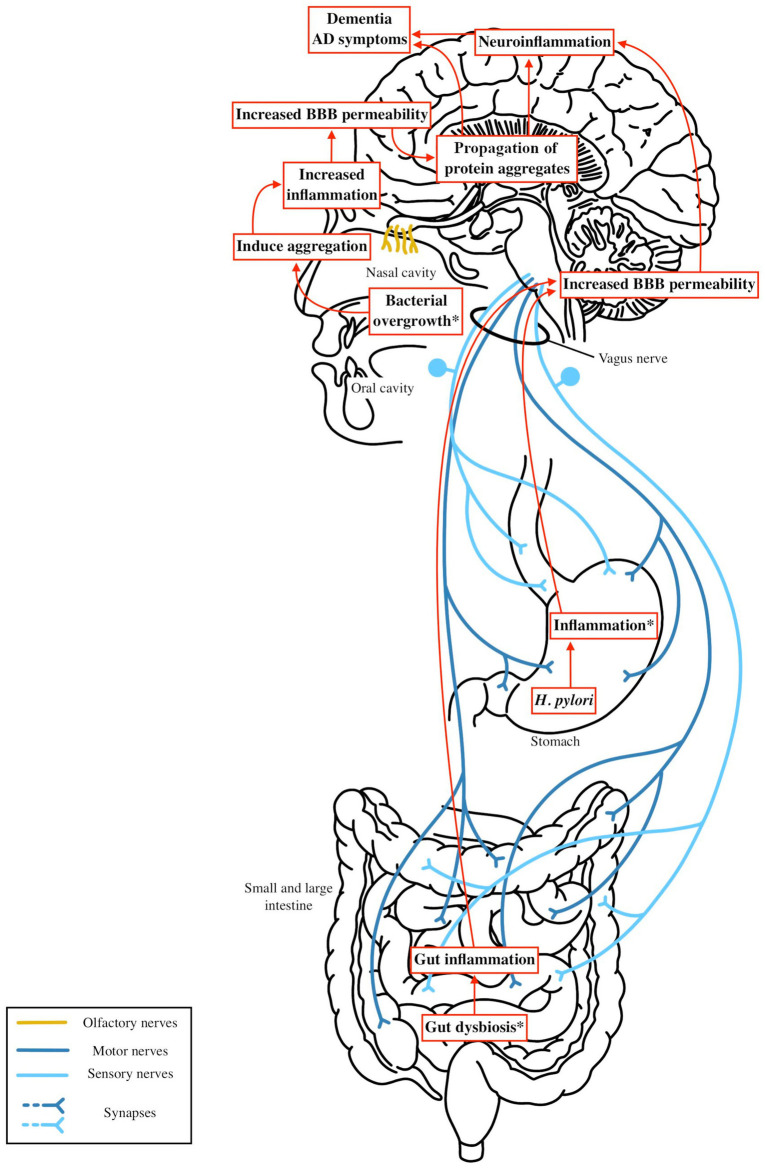
Microbiota dysbiosis—such as increased levels of LPS and reduced SCFA producing bacteria—or gastrointestinal infections like *H. pylori* or periodontal disease, can trigger gut and systemic inflammation. This inflammation activates systemic immune pathways that increase blood brain barrier (BBB) permeability. As the BBB becomes more permeable, the brain becomes more vulnerable to exogenous factors, including microbial metabolites or bacterial antigens, which may contribute to the aggregation of beta amyloid or tau. These aggregates could be initiated by microbial products from the nasal or oral cavity and spread to the brain via the olfactory or other cranial nerves. This process amplifies neuroinflammation in the central nervous system, accelerating neuronal damage and cognitive decline in Alzheimer’s disease.

The microbiota-gut-brain axis links gut microbial dysbiosis to Parkinson’s disease and Alzheimer’s disease pathogenesis through systemic inflammation, blood–brain barrier dysfunction, and protein aggregation. This section synthesizes findings on microbial shifts, metabolites (SCFAs and LPS) and neural pathways to propose mechanisms driving PD and AD, as illustrated in Figures 3, 4.

In PD, as outlined prior, the Braak hypothesis posits that α-syn pathology may originate in the gut, with aggregates spreading to the CNS via the vagus nerve. This is supported by evidence of α-syn aggregates in the ENS of PD patients, often preceding motor symptoms by years, and by the observation that truncal vagotomy reduces PD risk (Braak et al., 2003a; Svensson et al., 2015; Liu et al., 2017). Changes in the microbiome, specifically an increase in *Akkermansia* combined with a low fiber diet, may predispose patients to an unhealthy gut lining that is more susceptible to exogenous insult. Elevated levels of pathogenic forms of LPS can further promote endothelial damage and chronic gut level inflammation, as well as systemic inflammation through cytokine release into circulation or by enabling bacterial translocation across the compromised gut lining. With reductions in SCFA producing bacteria, particularly butyrate producers like *Roseburia*, this inflammation may go unchecked, leading to increased epithelial permeability and elevated systemic cytokine levels. Additionally, increased barrier permeability may expose endogenous α-syn to environmental factors such as curli amyloid or other bacterial metabolites that promote local oxidative stress, facilitating its aggregation and propagation along the gut lining. This aggregated α-syn can then spread to more proximal regions via the vagus or olfactory nerve, a process further exacerbated by systemic inflammation and reduced SCFA levels, which contribute to increased BBB permeability (Figure 3).

In AD, the Braak hypothesis posits tau pathology begins in the transentorhinal cortex, potentially initiated by oral/nasal microbial products via the olfactory bulb, as neurofibrillary tangles are found in the olfactory epithelium (Braak and Del Tredici, 2015; Shimizu et al., 2004). While there are currently no models demonstrating beta amyloid or tau pathology traveling from the gut to the brain in AD, similar routes of propagation via cranial nerves, particularly the olfactory nerve, warrant further exploration. Increased intestinal permeability and systemic inflammation may facilitate the spread or exacerbation of Aβ and tau pathology by compromising the BBB and promoting microglial activation. Microbial metabolites, such as cadaverine, may further disrupt cell signaling and exacerbate neuroinflammation (Amin et al., 2021). Gut dysbiosis in AD illustrated increases in proinflammatory taxa such as *Alistipes* and *Bacteroides*, a reduction in SCFA producing bacteria, and the presence of pathogenic species like *Porphyromonas gingivalis* and *Helicobacter pylori*. These microbial changes promote chronic gut and oral inflammation and contribute to systemic inflammation through mechanisms such as LPS mediated TLR4 activation. This inflammation may be more pronounced in individuals carrying the APOE ε4 allele, which enhances proinflammatory M1 microglial responses (Dias et al., 2025). These pathways are illustrated in Figure 4. Together, these mechanisms suggest that targeting gut inflammation and restoring a more anti-inflammatory microbial composition may be a viable strategy for slowing AD progression and mitigating neuroinflammatory damage.

## 4 Limitations and future directions

Understanding microbiome changes in PD and AD involves several limitations that shape our interpretations and guide future research. Fecal microbiome analyses have inherent limitations, as they provide only a snapshot of the gut microbiome and primarily reflect bacteria from the distal colon. This limitation is particularly relevant in PD research since the vagus nerve has been shown to have clear influence on pathogenesis, but vagal innervation ends around the splenic flexure. However, testing proximal microbiome profiles – between the esophagus and the transverse colon – is challenging due to the invasive nature of available procedures. Also, the microbial composition varies significantly along different segments of the GI tract, with distinct microbiota in the upper GI tract (e.g., stomach and duodenum) compared to the lower GI tract (e.g., colon and rectum). Additionally, microbiome sampling via colonoscopy may not accurately reflect the natural state of the colon due to pre-procedure preparation such as fasting and bowel cleansing.

For both AD and PD, sampling from the distal esophagus and proximal duodenum could provide valuable insights, especially given the role of *H. pylori* and other proximal infections in systemic inflammation and vagus nerve involvement. Endoscopic biopsies could enable such studies, but obtaining sufficient patient and control data presents a significant challenge.

AD microbiome research lags behind PD, with fewer studies and a focus on fecal data that may overlook oral or nasal influences on pathology. However, gut dysbiosis could still amplify AD’s neuroinflammation. Thus, fecal microbiome sampling remains valuable for identifying gut-derived factors that may exacerbate neuroinflammation and worsen AD progression by microglial cytokine release and increased BBB permeability. Additionally, significant associations between specific bacterial species or SCFAs may be overlooked because most studies focus on broad taxonomic classifications at the family or genus level. This review emphasizes the need for more precise bacterial analyses at the species or strain level to uncover novel pathological mechanisms. This is especially important since many bacteria previously thought to be beneficial, such as those in the genus *Akkermansia,* are shown to be increased. However, without concurrent metabolomic analysis, the functional implications of these microbial shifts remain unclear. Microbiome profiling alone provides limited insight into mechanistic pathways, as it does not capture the dynamic metabolic outputs of microbial communities. Integrating species-level with metabolome analysis is therefore essential to elucidate how specific bacterial strains and their metabolites influence host physiology and disease progression.

To definitively establish a causal link between specific microbial products and pathological protein aggregation or neuronal death in AD and PD models, improved species and metabolite-level analyses are needed, along with clearer experimental animal models that can directly follow protein aggregates. Approaches such as colonizing germ-free animal models with specific bacterial strains or profiles may help observe direct effects on neurodegenerative pathology. Additionally, administration or depletion of specific microbial metabolites—like short-chain fatty acids or lipopolysaccharides—can clarify their roles in modulating protein aggregation and neuroinflammation. Fecal microbiota transplantation experiments, whereby microbiota from diseased versus healthy subjects are transferred into recipient animals at different points of the digestive tract, may offer further insight into causative relationships. *In vitro* neuronal cultures exposed to microbial products can also provide mechanistic understanding of how bacteria influence protein misfolding and neuronal health. Despite these strategies, challenges remain, including the complexity of microbiome and nervous system, variability in host genetics and environment, and translatability between animal models and humans due to physiology and diet differences. Addressing these hurdles is critical for moving beyond correlation, enabling a clearer understanding of how specific microbial factors drive neurodegeneration, and ultimately guiding the development of microbiome-targeted therapies.

Furthermore, translating these findings into personalized microbiome-based therapies faces significant hurdles. Individual variability in host genetics, diet, and concurrent medications influence the microbiome and its metabolites, likely altering therapeutic outcomes. For instance, dietary habits can promote certain microbiota profiles, while medications such as antibiotics, anticholinergic drugs in AD, or dopaminergic treatments common in PD patients may disrupt microbial balance and gut transit time (Hays et al., 2024; Popescu et al., 2024; Menozzi and Schapira, 2024). Developing interventions to modulate systemic inflammation, blood–brain barrier integrity, or neural propagation in AD and PD will require a better understanding of how host-specific factors impact therapy design. This review aims to guide researchers toward specific bacterial species and microbial products that warrant more precise study, with the goal of developing therapies that foster microbiota profiles capable of reducing systemic inflammation and limiting the influence of external factors that may contribute to pathological protein aggregation.

## 5 Conclusion

The microbiota–gut–brain axis offers a promising framework for understanding the pathogenesis of Alzheimer’s disease and Parkinson’s disease. This review highlights how gut dysbiosis may contribute to aggregate pathology through its influence on BBB permeability, neuroinflammation, and aggregation pathways. These effects may be mediated by reductions in SCFA-producing bacteria and increases in bacteria with antigenic components like bacterial lipopolysaccharide. In PD, such microbial shifts are associated with *α*-synuclein propagation via the vagus nerve, while in AD, they appear to amplify systemic inflammation and may contribute to amyloid-*β*/tau pathology, potentially through olfactory or systemic immune routes. Shared mechanisms, such as LPS-driven inflammation and decreased butyrate levels, suggest overlapping therapeutic targets, while distinct microbial profiles (e.g., increased *Akkermansia* in PD) illustrate the importance of disease-specific strategies. By identifying potential microbiome-based biomarkers, including key bacterial taxa and metabolites, this review supports further research into early diagnostic tools and microbiota-targeted interventions, such as probiotics or dietary modification. Ongoing exploration of the microbiota–gut–brain axis will be essential for advancing medicine approaches to these complex neurodegenerative diseases.
