# Supplementary material for: Gut dysbiosis as a potential driver of Parkinson’s and Alzheimer’s disease pathogenesis
Source: Front Neurosci. 2025 Aug 13;19:1600148. doi: 10.3389/fnins.2025.1600148 (PMC12380846; doi:10.3389/fnins.2025.1600148)
Supplement: Supplementary file 1 [file Table_1.DOCX]

Supplementary Material

# Supplementary Tables

**Supplemental Table 1. Studies Showing Increased Abundance of Bacterial Families.** *Source with no asterisk denotes PD study, Source with asterisk** denotes AD study

| **Family** | **Sources Showing increased Abundance** |
| --- | --- |
| *Bacteroidaceae* | Keshavarzian et al., 2015; Vogt et al., 2017* |
| *Bifidobacteriaceae* | Lin et al., 2018; Zhao et al., 2018; Barichella et al., 2019; Li et al., 2019; Hill-Burns et al., 2017; Aho et al., 2019 |
| *Clostridiaceae* | Keshavarzian et al., 2015 |
| *Enterobacteriaceae* | Unger et al., 2016; Barichella et al., 2019; Pietrucci et al., 2019; Liu et al., 2018*; Toh et al., 2022* |
| *Enterococcaceae* | Lin et al., 2019; Pietrucci et al., 2019; Hopfner et al., 2017; Li et al., 2017; Zhuang et al., 2018* |
| *Eubacteriaceae* | Lin et al., 2018; Vascellari et al., 2020 |
| *Lactobacillaceae* | Zhao et al., 2018; Lin et al., 2019; Qian et al., 2020; Barichella et al., 2019; Pietrucci et al., 2019; Hill-Burns et al., 2017; Hasegawa et al., 2015; Tan et al., 2021; Hopfner et al., 2017; Zhuang et al., 2018* |
| *Oscillospiraceae/Ruminococcaceae* | Li et al., 2019; Li et al., 2020; Scheperjans et al., 2015; Zhuang et al., 2018* |
| *Rikenellaceae* | Li et al., 2019; Qian et al., 2018; Lin et al., 2019; Qian et al., 2020; Li et al., 2020; Vascellari et al., 2020; Aho et al., 2019; Vogt et al., 2017* |
| *Streptococcaceae* | Zhao et al., 2018; Qian et al., 2020; Vascellari et al., 2020; Li et al., 2017 |
| *Veillonellaceae* | Li et al., 2019; Zhao et al., 2018; Li et al., 2017; Liu et al., 2018* |
| *Verrucomicrobiaceae* | Li et al., 2019; Lin et al., 2019; Bedarf et al., 2017; Heintz-Buschart et al., 2018; Barichella et al., 2019; Vascellari et al., 2020; Keshavarzian et al., 2015; Hill-Burns et al., 2017; Scheperjans et al., 2015; Tan et al., 2021; Lubomski et al., 2022 |

**Supplemental Table 2. Studies Showing Decreased Abundance of Bacterial Families.** *Source with no asterisk denotes PD study, Source with asterisk** denotes AD study

| **Family** | **Sources Showing Decreased Abundance** |
| --- | --- |
| *Bacteroidaceae* | Vascellari et al., 2020; Zhuang et al., 2018* |
| *Bifidobacteriaceae* | Vogt et al., 2017 |
| *Clostridiaceae* | Vogt et al., 2017; Liu et al., 2018; Hung et al., 2022 |
| *Enterococcaceae* | Unger et al., 2016 |
| *Lachnospiraceae* | Lin et al., 2018; Bedarf et al., 2017; Cosma-Grigorov et al., 2020; Barichella et al., 2019; Pietrucci et al., 2019; Vascellari et al., 2020; Keshavarzian et al., 2015; Hill-Burns et al., 2017; Aho et al., 2019; Cirstea et al., 2020; Liu et al., 2018*; Veehaar et al., 2022*; Zhuang et al., 2018*; Hung et al., 2022* |
| *Lactobacillaceae* | Li et al., 2019; Qian et al., 2018; Unger et al., 2016; Veehaar et al., 2022* |
| *Oscillospiraceae/Ruminococcaceae* | Vogt et al., 2017*; Liu et al., 2018*; Ueda et al., 2021* |
| *Prevotellaceae* | Lin et al., 2019; Li et al., 2020; Unger et al., 2016; Bedarf et al., 2017; Scheperjans et al., 2015; Aho et al., 2019 |
| *Rikenellaceae* | Hung et al., 2022* |
| *Sphingobacteriaceae* | Vascellari et al., 2020 |
| *Streptococcaceae* | Lin et al., 2018; Bedarf et al., 2017 |
| *Veillonellaceae* | Lubomski et al., 2022; Zhuang et al., 2018* |

**Supplemental Table 3.** Comprehensive List of Studies Showing Increase or Decrease in Specific Bacterial Genus. *Source with no asterisk denotes PD study, Source with asterisk** denotes AD study.

| **Genus** | **Increased (Source)** | **Decreased (Source)** |
| --- | --- | --- |
| *Acidaminococcus* | Zhao et al., 2018; Baldini et al., 2020; Li et al., 2017; Toh et al., 2022 | - |
| *Acinetobacter* | Qian et al., 2018; Zhao et al., 2018; Li et al., 2017 | - |
| *Adlercreutzia* | - | Vogt et al., 2017*; Cammann et al., 2023* |
| *Akkermansia* | Li et al., 2019; Lin et al., 2019; Li et al., 2020; Zhang et al., 2020; Bedarf et al., 2017; Heintz-Buschart et al., 2018; Barichella et al., 2019; Vascellari et al., 2020; Keshavarzian et al., 2015; Tan et al., 2021; Baldini et al., 2020; Lubomski et al., 2022; Toh et al., 2022 | - |
| *Alistipes* | Lin et al., 2018; Li et al., 2019; Qian et al., 2018; Qian et al., 2020; Li et al., 2020; Toh et al., 2022; Vogt et al., 2017*; Cammann et al., 2023*; Haran et al., 2019*; Hung et al., 2022* | Hung et al., 2022* |
| *Anaerostipes* | Vascellari et al., 2020 | Cammann et al., 2023*; Ueda et al., 2021* |
| *Anaerotruncus* | Heintz-Buschart et al., 2018; Baldini et al., 2020 | - |
| *Aquabacterium* | Qian et al., 2018 | - |
| *Bacteroides* | Keshavarzian et al., 2015; Vogt et al., 2017*; Cammann et al., 2023*; Haran et al., 2019*; Hung et al., 2022* | Vascellari et al., 2020; Petrov et al., 2017; Zhuang et al., 2018* |
| *Barnesiella* | Haran et al., 2019* | - |
| *Bifidobacterium* | Lin et al., 2018; Li et al., 2020; Unger et al., 2016; Barichella et al., 2019; Vascellari et al., 2020; Hill-Burns et al., 2017; Aho et al., 2019; Tan et al., 2021; Cirstea et al., 2020; Petrov et al., 2017; Toh et al., 2022* | Vogt et al., 2017* |
| *Bilophila* | Lin et al., 2018; Lin et al., 2019; Cirstea et al., 2020; Baldini et al., 2020; Vogt et al., 2017* | - |
| *Blautia* | Vogt et al., 2017* | Lin et al., 2018; Zhao et al., 2018; Vascellari et al., 2020; Keshavarzian et al., 2015; Hill-Burns et al., 2017; Aho et al., 2019; Li et al., 2017; Liu et al., 2018* |
| *Brevibacterium* | - | Lin et al., 2018; Vascellari et al., 2020 |
| *Brevundimonas* | - | Lin et al., 2018 |
| *Bulleidia* | - | Lin et al., 2018 |
| *Butyricicoccus* | Qian et al., 2018 | Lubomski et al., 2022 |
| *Butyricimonas* | Lin et al., 2019; Toh et al., 2022 | - |
| *Butyrivibrio* | Li et al., 2020; Tan et al., 2021 | Vascellari et al., 2020; Toh et al., 2022; Haran et al., 2019* |
| *Caldicellulosiruptor* | Vascellari et al., 2020 | - |
| *cc115* | - | Vogt et al., 2017* |
| *Christensenella/Catabacter* | Tan et al., 2021; Petrov et al., 2017; Baldini et al., 2020; Toh et al., 2022 | - |
| *Citrobacter* | Pietrucci et al., 2019 | - |
| *Cloacibacillus* | Tan et al., 2021; Toh et al., 2022 | - |
| *Clostridium* | Qian et al., 2018; Heintz-Buschart et al., 2018; Vascellari et al., 2020 | Aho et al., 2019; Vogt et al., 2017* |
| *Clostridium XVIII* | Qian et al., 2018 | - |
| *Clostridium IV* | Qian et al., 2018 | - |
| *Collinsella* | Cirstea et al., 2020; Toh et al., 2022; Cammann et al., 2023* | - |
| *Coprococcus* | Li et al., 2019 | Vascellari et al., 2020; Keshavarzian et al., 2015 |
| *Desulfovibrio* | Lin et al., 2018; Qian et al., 2018; Vascellari et al., 2020; Toh et al., 2022 | - |
| *Dialister* | Lin et al., 2018 | Vogt et al., 2017* |
| *Dorea* | - | Keshavarzian et al., 2015; Petrov et al., 2017 |
| *Eikenella* | - | Lin et al., 2018 |
| *Eisenbergiella* | - | Cammann et al., 2023* |
| *Enterobacter* | Qian et al., 2020; Vascellari et al., 2020 | - |
| *Enterococcus* | Zhao et al., 2018; Lin et al., 2019; Pietrucci et al., 2019; Li et al., 2017 | - |
| *Escherichia/Shigella* | Zhao et al., 2018; Pietrucci et al., 2019; Vascellari et al., 2020; Li et al., 2017; Toh et al., 2022; Cattaneo et al., 2017* | - |
| *Eubacterium* | - | Bedarf et al., 2017; Cammann et al., 2023*; Ueda et al., 2021* |
| *Faecalibacterium* | Toh et al., 2022 | Lin et al., 2018; Zhao et al., 2018; Lin et al., 2019; Weis et al., 2019; Kidd and Schneider, 2010; Hill-Burns et al., 2017; Petrov et al., 2017; Lubomski et al., 2022; Li et al., 2017; Ueda et al., 2021* |
| *Fusicatenibacter* | - | Weis et al., 2019; Heintz-Buschart et al., 2018; Lubomski et al., 2022 |
| *Fusobacterium* | Zhang et al., 2020 | - |
| *Gemella* | Vogt et al., 2017* | - |
| *Haemophilus* | - | Lin et al., 2018; Zhao et al., 2018; Lubomski et al., 2022 |
| *Halomonas* | - | Lin et al., 2018 |
| *Hyphomonas* | - | Lin et al., 2018 |
| *Intestinibacter* | - | Cammann et al., 2023* |
| *Klebsiella* | Qian et al., 2018; Pietrucci et al., 2019 | Vascellari et al., 2020 |
| *Lachnobacterium* | Lin et al., 2018 | - |
| *Lachnoclostridium* | - | Veehaar et al., 2022*; Zhuang et al., 2018*; Haran et al., 2019* |
| *Lachnospira* | Li et al., 2019; Cammann et al., 2023* | Lin et al., 2018; Vascellari et al., 2020 |
| *Lachnospiraceae incertae sedis* | Qian et al., 2018 | - |
| *Lachnospiraceae UCG-001* | Li et al., 2019 | - |
| *Lachnospiraceae UCG-008* | - | Cammann et al., 2023* |
| *Unclassified Lachnospiraceae* | - | Hill-Burns et al., 2017; Toh et al., 2022 |
| *Lactobacillus* | Lin et al., 2019; Qian et al., 2020; Li et al., 2020; Pietrucci et al., 2019; Hill-Burns et al., 2017; Tan et al., 2021; Hasegawa et al., 2015; Petrov et al., 2017; Baldini et al., 2020; Toh et al., 2022 | Li et al., 2019; Qian et al., 2018 |
| *Megamonas* | Zhao et al., 2018; Li et al., 2017 | - |
| *Megasphaera* | Li et al., 2017; Toh et al., 2022 | Zhao et al., 2018 |
| *Methylobacterium* | - | Lin et al., 2018 |
| *Mitsuokella* | - | Zhao et al., 2018 |
| *Morganella* | Lin et al., 2018 | - |
| *Mucispirillum* | Lin et al., 2019 | - |
| *Mycoplana* | - | Lin et al., 2018 |
| *Ochrobactrum* | - | Lin et al., 2018 |
| *Odoribacter* | Lin et al., 2019; Haran et al., 2019 | - |
| *Oscillibacter* | - | Cammann et al., 2023* |
| *Oscillospira* | Zhang et al., 2020; Barichella et al., 2019; Keshavarzian et al., 2015; Petrov et al., 2017 | - |
| *Parabacteroides* | Li et al., 2019; Lin et al., 2019; Barichella et al., 2019 | Vascellari et al., 2020 |
| *Paraprevotella* | Qian et al., 2018 | - |
| *Pedobacter* | - | Lin et al., 2018 |
| *Peptoclostridium* | Li et al., 2020 | - |
| *Peptoniphilus* | Weis et al., 2019 | - |
| *Peptostreptococcus* | - | - |
| *Phascolarctobacterium* | Li et al., 2019; Vogt et al., 2017*; Hung et al., 2022* | - |
| *Prevotella* | Ueda et al., 2021* | Lin et al., 2019; Li et al., 2020; Bedarf et al., 2017; Aho et al., 2019; Petrov et al., 2017; Cammann et al., 2023* |
| *Prosthecobacter* | Vascellari et al., 2020 | - |
| *Proteus* | Zhao et al., 2018; Li et al., 2017 | - |
| *Pseudidiomarina* | - | Lin et al., 2018 |
| *Pseudobutyrivibrio* | - | Vascellari et al., 2020 |
| *Pseudochrobactrum* | - | Lin et al., 2018 |
| *Ralstonia* | Lin et al., 2018 | - |
| *Roseburia* | Li et al., 2019 | Lin et al., 2018; Barichella et al., 2019; Pietrucci et al., 2019; Vascellari et al., 2020; Keshavarzian et al., 2015; Hill-Burns et al., 2017; Aho et al., 2019; Cirstea et al., 2020; Lubomski et al., 2022; Toh et al., 2022; Cammann et al., 2023* |
| *Rubellimicrobium* | - | Lin et al., 2018 |
| *Ruminococcus* | Li et al., 2019 | Zhao et al., 2018; Barichella et al., 2019; Li et al., 2017; Liu et al., 2018*; Ueda et al., 2021* |
| *Unclassified Ruminococcaceae* | - | Bedarf et al., 2017 |
| *Salmonella* | Pietrucci et al., 2019 | - |
| *Sediminibacterium* | - | Qian et al., 2018 |
| *Serratia* | Vascellari et al., 2020 | - |
| *Slackia* | Vascellari et al., 2020 | - |
| *SMB53* | - | Vogt et al., 2017* |
| *Sphingobacterium* | - | Lin et al., 2018 |
| *Sphingomonas* | Qian et al., 2018 | - |
| *Streptococcus* | Zhao et al., 2018; Qian et al., 2020; Vascellari et al., 2020; Baldini et al., 2020; Li et al., 2017; Toh et al., 2022 | Lin et al., 2018; Li et al., 2019; Lubomski et al., 2022 |
| *Stenotrophomonas* | - | Lin et al., 2018 |
| *Subdoligranulum* | Zhuang et al., 2018* | - |
| *Succinatimonas* | - | Lin et al., 2018 |
| *Sutterella* | Cammann et al., 2023* | Vascellari et al., 2020 |
| *Turicibacter* | Baldini et al., 2020 | Vogt et al., 2017* |
| *Veillonella* | Lin et al., 2019; Vascellari et al., 2020; Toh et al., 2022; Cammann et al., 2023* | - |
